# In-hospital triggers of takotsubo syndrome: a case report on witnessing sudden death in a hospital roommate

**DOI:** 10.1093/ehjcr/ytad556

**Published:** 2023-11-09

**Authors:** Assem Aweimer, Ibrahim El-Battrawy, Patrick Beck, Andreas Mügge

**Affiliations:** Bergmannsheil Bochum, Medical Clinic II, Department of Cardiology and Angiology, Ruhr University Bochum, Bürkle-de-la-Camp-Platz 1, 44789 Bochum, Germany; Bergmannsheil Bochum, Medical Clinic II, Department of Cardiology and Angiology, Ruhr University Bochum, Bürkle-de-la-Camp-Platz 1, 44789 Bochum, Germany; Bergmannsheil Bochum, Medical Clinic II, Department of Cardiology and Angiology, Ruhr University Bochum, Bürkle-de-la-Camp-Platz 1, 44789 Bochum, Germany; Bergmannsheil Bochum, Medical Clinic II, Department of Cardiology and Angiology, Ruhr University Bochum, Bürkle-de-la-Camp-Platz 1, 44789 Bochum, Germany

**Keywords:** Case report, Takotsubo syndrome, Stress, In-hospital mortality

## Abstract

**Background:**

Takotsubo syndrome (TTS) is characterized by acute left ventricular dysfunction, mimicking an acute myocardial infarction, in the absence of obstructed coronary arteries. It is often triggered by physical or emotional stress, with catecholamines playing a central role in its pathophysiology. Recent advances have been made in categorizing TTS patients based on trigger events and comorbidities, as well as proposed classifications differentiating primary and secondary TTS. In-hospital triggers for (secondary) TTS appear to be quite common, and our aim is to bring attention of this prevalent phenomenon.

**Case summary:**

We present the clinical course of an 80-year-old man who developed TTS after witnessing the sudden death of his roommate during his hospital stay. Initially hospitalized for bradycardia and complete atrioventricular block, the patient was discharged after a pacemaker implantation. However, he returned to the hospital 3 days later with chest pain and other symptoms indicative of TTS. Diagnostic tests confirmed apical ballooning consistent with TTS, and subsequent echocardiograms showed a substantial improvement in left ventricular function.

**Discussion:**

The case is classified as in-hospital TTS, occurring unexpectedly during medical care, and suggests that secondary TTS could represent a certain ‘basic risk’ for hospitalized patients. We want to emphasize the importance of reducing pain and fear in the hospital setting and encourage further research to understand the association between TTS and medical procedures and therapies. Overall, this case underscores the need for strategies to reduce the frequency of TTS in hospitalized patients.

**Learning points**

- It is important to realize that pain and fear in the hospital setting during medical procedures and therapies could trigger an in-hospital takotsubo syndrome event.
- To the greatest extent possible, efforts should be made to prevent patients from directly witnessing emergency situations involving other patients.

## Introduction

Takotsubo syndrome (TTS), initially named as tako-tsubo-like left ventricular (LV) dysfunction by Sato *et al.*,^1^ is characterized by an acute onset of dyskinetic ‘ballooning’ in the apical region of the left ventricle, with hyperkinetic basal segments, and in the absence of obstructed coronary arteries, mimicking an acute myocardial infarction.^2^ Frequently, this acute syndrome is preceded by physical or emotional stress or other conditions associated with high catecholaminergic levels; thus, catecholamines may play a central role in the pathophysiology of this syndrome.^3,4^

In most cases, this acute syndrome is reversible, but serious complications may occur, including cardiogenic shock, ventricular wall rupture, thrombi formation, or arrhythmias, leading to an all-cause death rate of 5.6% per patient-year.^2,3,5^

A variety of ‘stressful events’ have been reported as triggers. Witnessing the death of a family member, co-worker, or stranger is an obvious stress trigger, but only five case reports have been published on this topic so far. Doesch *et al.*^6^ report on a 60-year-old woman, witnessing the sudden cardiac death of a co-worker, who recovered quickly within 18 h. Lisung *et al.*^7^ report on a woman in her early 70s with stress-induced cardiomyopathy after her husband suffered from out-of-hospital cardiac arrest and died after hospital admission. The woman could be discharged after 48 h. Finsterer and Stollberger^8^ report on a 64-year-old man who took part in a funeral for his brother-in-law. At admission, he presented with global amnesia and TTS, from which he recovered uneventfully. Shah and Cummings^9^ report on a 60-year-old female who learned that a friend passed away unexpectedly. She was discharged on Day 2 after hospital admission. To *et al.*^10^ report on a 60-year-old woman who witnessed the uneventful resuscitation of her husband. She could be discharged in a stable condition.

We report the clinical course of an 80-year-old man who witnessed the sudden death of a roommate during his hospital stay.

## Summary figure

**Table:** 

Time	Events
Day 0	Admission to emergency department with complete AV block, normal systolic LV function in transthoraic echocardiography
Day 1	Pacemaker Implantation
Day 3	Hospital discharge
Day 6	Re-admission with chest pain interpreted as an acute coronary syndrome, coronary angiography and levocardiography with the evidence of apical ballooning
Day 10	Improvement of systolic LV function in transthoraic echocardiography
Day 41	Restoration of normal systolic LV function in transthoraic echocardiography

## Case presentation

The patient presented to the emergency department due to exercise intolerance and dyspnoea. The electrocardiogram (ECG) revealed severe bradycardia with complete atrioventricular block. The patient had a medical history of diabetes, arterial hypertension, and Parkinson’s disease. Transthoracic echocardiography revealed no evidence of structural heart disease, with normal systolic pump function and no wall motion abnormalities. Consequently, a two-chamber pacemaker implantation was performed without any complications. The patient did not require any vasopressors or catecholamines during sedation, and the local anaesthetic used was free from adrenaline. After an overall 3-day hospital stay, the patient was discharged.

**Figure 1:**
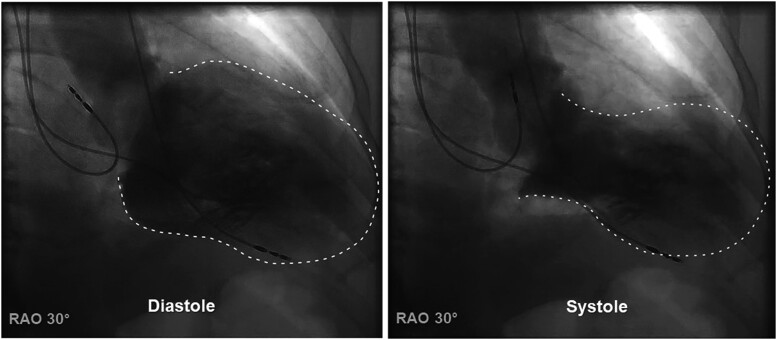
Levocardiography with typical pattern of apical ballooning in TTS. RAO, right anterior oblique.

Three days later, the patient returned to the emergency department with chest pain, dizziness, and cold sweating. Upon inquiry, the patient reported that the pressure sensation had first occurred several hours after witnessing a resuscitation procedure following the sudden cardiac death of his roommate during the last hospital stay, which was at the morning after pacemaker implantation. However, the sensation had now acutely worsened. The ECG showed continuous ventricular pacing, and laboratory findings revealed a significant elevation in troponin levels (see Supplementary material online). An echocardiogram revealed akinesia of the apex with moderately reduced LV systolic function. Except for an intermediate non–intervention-requiring stenosis in the right coronary artery, coronary atherosclerosis was observed without significant stenoses. Levocardiography also confirmed apical akinesia consistent with apical ballooning (*Figure 1*). Due to the recent pacemaker implantation, a magnetic resonance imaging (MRI) could not be performed. A follow-up echocardiogram after several days showed a substantial improvement in systolic LV function, suggesting a diagnosis of TTS. Five weeks later, a follow-up echocardiogram showed complete normalization of global systolic LV function.

## Discussion

Our case fulfils the criteria for TTS, which have been recently outlined by a task force of the European Society of Cardiology (ESC) Heart Failure Association.^11^ Several advances have been made in the past to classify TTS patients according to the trigger event, circumstances, and comorbidities. Recently, we observed in a small multicentre observational study a high proportion of abnormal thyroid function and suggested a categorization of TTS patients in ‘endocrine-type’ response and ‘stress-type’ response.^12^ The task force suggests differentiating a primary from a secondary type of TTS.

The primary type encompasses patients with acute cardiac symptoms as the primary reason for seeking medical care, e.g. becoming hospitalized. The secondary type includes patients who are already hospitalized for various medical, obstetric, surgical, or psychiatric reasons other than TTS and are exposed within the hospital to an excess of catecholamines and/or a stressful event. Isogai *et al.*^13^ differentiated between in-hospital and out-of-hospital TTS, identifying 419 patients with in-hospital–acquired TTS, usually secondary to medical illness after hospital admission, and 3300 patients with out-of-hospital–acquired TTS occurring after a stressful event during 2010 and 2013 in a Japanese database. Of note, the in-hospital mortality rate was significantly higher in patients with in-hospital–acquired TTS (17.9% vs. 5.4%; *P* < 0.001), even after adjustment for patient background.

Núñez-Gil *et al.*,^14^ on behalf of the RETAKO Investigators, differentiated between psychic and physical triggers and analysed a local and national registry on TTS (2003–13) with 338 patients. They noted that the majority (*n* = 265) witnessed significant psychic stress and defined this as primary TTS, in contrast to secondary TTS with triggers related to ‘physical’ factors such as surgery or trauma. Patients with secondary TTS had a worse short- and long-term prognosis in terms of mortality, recurrence, and readmissions.

In a recent editorial, Galiuto and Crea^15^ proposed a novel classification of TTS based on several items, including the type of trigger, the absence or presence of organic disease, and the severity and reversibility of cardiac dysfunction. Their primary type includes postmenopausal women with emotional stress as the trigger, showing early recovery and a good prognosis. According to their literature research, they suggest that this type of TTS has a microvascular dysfunction as the underlying pathophysiological mechanism. The secondary type includes men and women with pre-existing organic diseases, accompanied by coronary artery disease, severe LV dysfunction, and late recovery. This type appears to be associated with a worse prognosis, and the underlying pathophysiological mechanism includes myocardial damage. However, the pathophysiology of TTS is not quite clear, as suggested by the proposal of Galiuto and Crea.^15^ Furthermore, these definitions have a large overlap, but they nevertheless classify distinct different groups. Unifying these classifications may be challenging.

Another consideration in TTS involves our case, which is characterized by an in-hospital occurrence where the patient unexpectedly faced acute stressors. The clinical course was uneventful, and the patient exhibited a rapid recovery without initiating any specific treatment. It may be of interest that all cases reported on this topic showed an uneventful clinical course and fast recovery. Based on the definitions of the ESC Heart Failure Association Taskforce, our case could also be classified as a secondary TTS. We wondered how frequently hospitalized patients experience TTS occurring unintentionally and unexpectedly, rather than being caused by severe underlying diseases, such as acute brain injuries, critical illness, sepsis, life-threatening conditions, or exposure to excessive catecholamines in the setting of a pheochromocytoma or anaphylactic shock therapy. In this case, we also have to take into account that the pacemaker implantation performed could potentially act as a stressor for TTS. This is in accordance with recent literature and numerous case reports that have observed TTS occurring after pacemaker implantation.^16^

**Figure 2:**
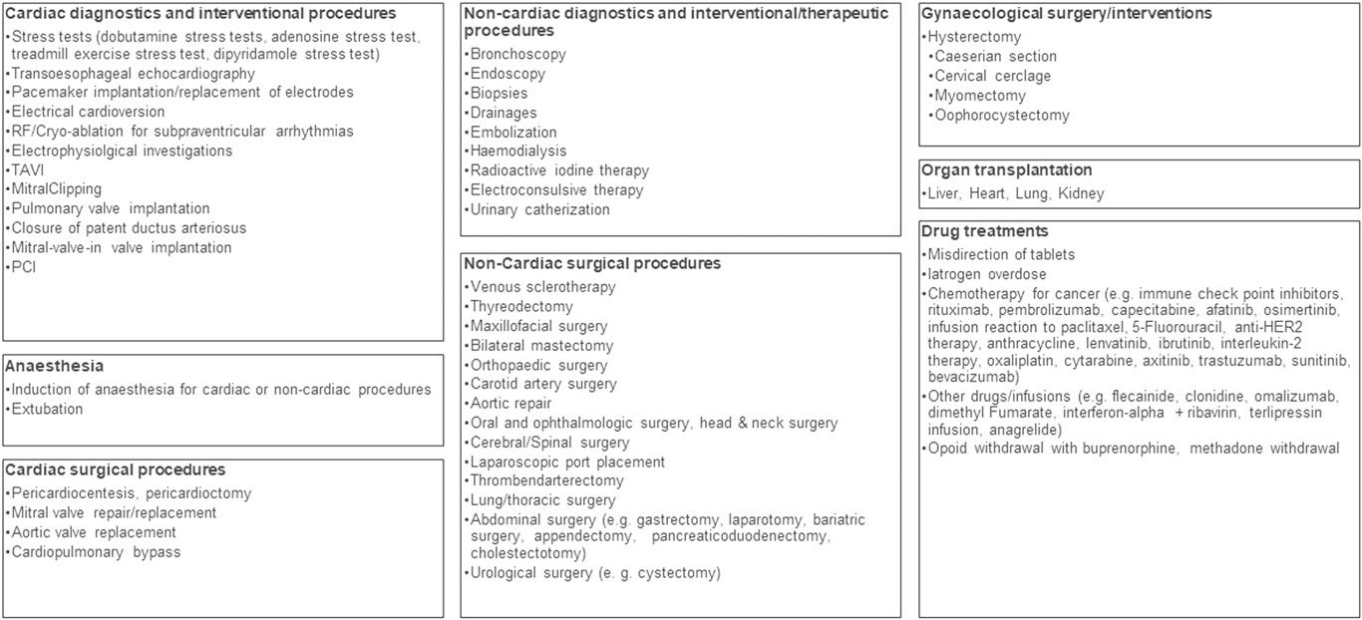
Triggers and circumstances for nosocomial TTS. RF, radiofrequency; TAVI, transfemoral aortic valve implantation; PCI, percutaneous coronary intervention.

In a literature search (PubMed search term ‘takotsubo’; date 24 May 2023), we noted numerous publications on in-hospital patients with TTS following regular diagnostic or therapeutic procedures or therapies. We found at least 542 out of 6541 publications (8.3%) with case reports or small case series addressing this subgroup of secondary TTS. *Figure 2* illustrates the manifold triggers and circumstances (no claim of completeness). There do not exist data on the incidence of unintended and unexpected TTS in hospitalized patients, and the true incidence may be obscure since a transient cardiac problem following a procedure/therapy may not always be clarified. It appears that secondary TTS could complicate virtually all hospital-related medical activities and may represent a certain ‘basic risk’ for hospitalized patients. This basic risk reminds us to use the term ‘nosocomial’ in this context. In parallel to nosocomial pneumonia, it seems reasonable to consider general strategies to reduce the frequency of this complication. We do not have a ready concept, but we would like to emphasize the importance of pain and fear reduction in the hospital setting and encourage further research to understand the association between TTS and certain medical procedures and therapies.

## Supplementary Material

ytad556_Supplementary_Data

## Data Availability

The authors confirm that the data supporting the findings of this case report are available within the manuscript and its online Supplementary material. Further details can be requested by contacting the corresponding author.

## Lead author biography

Dr Assem Aweimer is a consultant cardiologist at BG University Hospital Bergmannsheil at Ruhr University Bochum. His clinical focus is rhythmology including electrophysiology and device therapy. In research, he primarily dedicates his efforts to takotsubo cardiomyopathy, particularly in relation to interactions with thyroid metabolism.

## Notes

**Consent:** The authors confirm that written consent for submission and publication of this case report including images and associated text has been obtained from the patient in line with COPE guidance.

**Funding:** None declared.

## References

[1] Sato TH , Uchida T, Dote KMI. Tako-tsubo-like left ventricular dysfunction due to multivessel coronary spasm. In: Kodama K Haze K and Hori M (eds.), Clinical Aspect of Myocardial Injury: From Ischemia to Heart Failure. Tokyo, Japan: Kagakuhyoronsha Publishing Co; 1990. p56–64.

[2] Singh T , Khan H, Gamble DT, Scally C, Newby DE, Dawson D. Takotsubo syndrome: pathophysiology, emerging concepts and clinical implications. Circulation 2022;145:1002–1019. 35344411 10.1161/CIRCULATIONAHA.121.055854 PMC7612566

[3] Couch LS , Channon K, Thum T. Molecular mechanisms of takotsubo syndrome. Int J Mol Sci 2022;23:12262. 36293121 10.3390/ijms232012262 PMC9603071

[4] Omerovic E , Citro R, Bossone E, Redfors B, Backs J, Bruns B, et al. Pathophysiology of takotsubo syndrome-a joint scientific statement from the Heart Failure Association Takotsubo Syndrome Study Group and Myocardial Function Working Group of the European Society of Cardiology—part 1: overview and the central role for catecholamines and sympathetic nervous system. Eur J Heart Fail 2022;24:257–273. Epub 2022 Feb 16. PMID: 34907620. 10.1002/ejhf.2400 34907620

[5] El-Battrawy I , Santoro F, Stiermaier T, Möller C, Guastafierro F, Novo G, et al Prevalence, management, and outcome of adverse rhythm disorders in takotsubo syndrome: insights from the international multicenter GEIST registry. Heart Fail Rev 2020;25:505–511.PMID: 31713085. 31713085 10.1007/s10741-019-09856-4

[6] Doesch C , Burgstahler C, Seeger A, Miller S, May AE. Chest pain and reversible midventricular ballooning in a woman after witnessing sudden cardiac death: a possible variant of takotsubo cardiomyopathy. Can J Cardiol 2009;25:e22. 19148349 10.1016/s0828-282x(09)70030-3 PMC2691890

[7] Lisung FG , Shah AB, Levitt HL, Coplan NB. Stress-induced cardiomyopathy. BMJ Case Rep 2015;2015:bcr2014208690.PMID: 25858931; PMCID: PMC4401914. 10.1136/bcr-2014-208690 PMC4401914 25858931

[8] Finsterer J , Stollberger C. Simultaneous transient global amnesia and takotsubo syndrome after death of a relative: a case report. J Med Case Rep 2019;13:22. 30678717 10.1186/s13256-018-1928-0 PMC6346500

[9] Shah A , Cummings ED. A women with chest pain. JACEP Open 2022;3:e12848. 36397939 10.1002/emp2.12848 PMC9664087

[10] To M , Zhang Y, Tompkins A, Chen R, Patankar K, Sangodkar S. A broken heart after witnessing a dying heart: a case of takotsubo cardiomyopathy. Cureus 2022;14:e28752. 36211117 10.7759/cureus.28752 PMC9529233

[11] Lyon AR , Bossone E, Schneider B, Sechtem U, Citro R, Underwood SR, et al Current state of knowledge on Takotsubo syndrome: a position statement from the Taskforce on Takotsubo Syndrome of the Heart Failure Association of the European Society of Cardiology. Eur J Heart Fail 2016;18:8–27.Epub 2015 Nov 9. PMID: 26548803. 10.1002/ejhf.424 26548803

[12] Aweimer A , El-Battrawy I, Akin I, Borggrefe M, Mügge A, Patsalis PC, et al Abnormal thyroid function is common in takotsubo syndrome and depends on two distinct mechanisms: results of a multicentre observational study. J Intern Med 2021;289:675–687. 33179374 10.1111/joim.13189

[13] Isogai T , Yasunaga H, Matsui H, Tanaka H, Ueda T, Horiguchi H, et al Out-of-hospital versus in-hospital Takotsubo cardiomyopathy: analysis of 3719 patients in the Diagnosis Procedure Combination database in Japan. Int J Cardiol 2014;176:413–417.Epub 2014 Aug 1. PMID: 25115252. 25115252 10.1016/j.ijcard.2014.07.110

[14] Núñez-Gil IJ , Almendro-Delia M, Andrés M, Sionis A, Martin A, Bastante T, et al Secondary forms of Takotsubo cardiomyopathy: a whole different prognosis. Eur Heart J Acute Cardiovasc Care 2016;5:308–316.Epub 2015 Jun 4. PMID: 26045512. 26045512 10.1177/2048872615589512

[15] Galiuto L , Crea F. Primary and secondary takotsubo syndrome: pathophysiological determinant and prognosis. Eur Heart J Acute Cardiovasc Care 2020;9:690–693.PMID: 33222496. 33222496 10.1177/2048872620963493

[16] Wei ZH , Dai Q, Wu H, Song J, Wang L, Xu B. Takotsubo cardiomyopathy after pacemaker implantation. J Geriatr Cardiol 2018;15:246–248.PMID: 29721007; PMCID: PMC5919816. 29721007 10.11909/j.issn.1671-5411.2018.03.010 PMC5919816

