# Supplementary figures and images for: In-hospital triggers of takotsubo syndrome: a case report on witnessing sudden death in a hospital roommate
Source: Eur Heart J Case Rep. 2023 Nov 9;7(12):ytad556. doi: 10.1093/ehjcr/ytad556 (PMC10691651; doi:10.1093/ehjcr/ytad556)

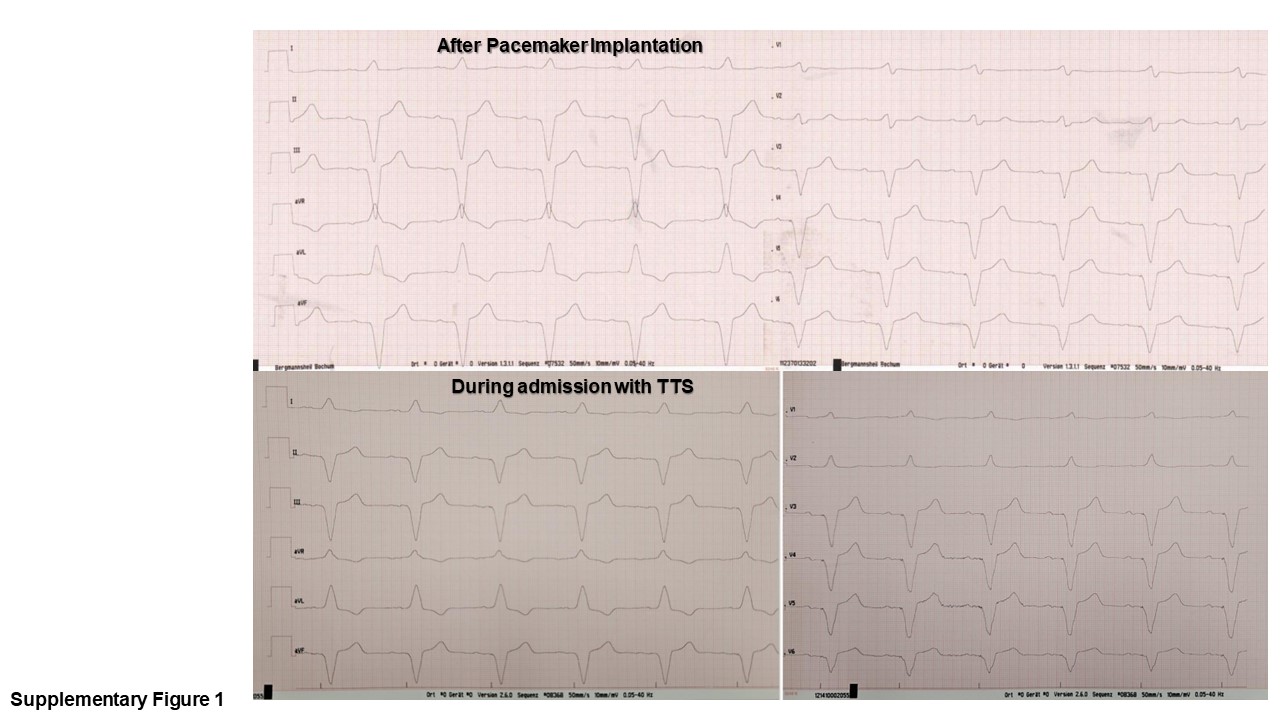

Supplement: ytad556_Supplementary_Data [file ytad556_supplementary_data.zip › Suppl Figure 1.jpg]
